# Interleukin-6 as A Prognostic Biomarker in Perinatal Asphyxia 

**DOI:** 10.22037/ijcn.v15i3.21773

**Published:** 2021

**Authors:** Hassan BOSKABADI, Gholamali MAAMOURI, Maryam ZAKERIHAMIDI, Fatemeh BAGHERI, Baratali MASHKANI, Shahin MAFINEJAD, Rahelah FARAMARZI, Abbas BOSKABADI, Ezzat KHODASHENAS, Elaheh HEIDARI, Forough RAKHSHANIZADEH

**Affiliations:** 1 Department of Pediatrics, Faculty of Medicine, Mashhad University of Medical Sciences, Mashhad, Iran.; 2 Department of Midwifery, School of Medical Sciences, Islamic Azad University, Tonekabon Branch, Tonekabon, Iran; 3 Department of Nursing, School of Nursing and Midwifery, Mashhad Branch, Islamic Azad University, Mashhad, Iran.; 4 Bioinformatics Research Group, Mashhad University of Medical Sciences, Mashhad. Iran.; 5 Department of Pediatrics, Faculty of Medicine, North Khorasan University of Medical Sciences, Iran

**Keywords:** Perinatal Asphyxia, Hypoxic Ischemic Encephalopathy, Interleukin-6 (IL-6), Developmental delay

## Abstract

**Objective:**

Early diagnosis is has a crucial role in both prevention and treatment of asphyxia-related complications. The current study aimed to evaluate the prognostic value of interleukin-6 (IL-6) and hypoxic-ischemic encephalopathy grade in the prediction of mortality and the developmental status of neonates affected by prenatal asphyxia.

**Materials & Methods:**

This cohort study was conducted on 38 term asphyxiated infants at Ghaem hospital, Mashhad, Iran, from 2013 to 2017. The HIE grade and serum IL-6 levels were determined at the time of birth. The developmental status was evaluated using the Denver II test at the end of the two-year follow-up.

**Results:**

HIE grade 3 resulted in 83% mortality rate and developmental delay among all survivors. The mean IL-6 level was 2.7 ng/ml in the control group (not affected HIE), which increased up to 29, 175, and 136 ng/ml in those with HIE grades of 1, 2, and 3, respectively. According to the ROC curve analysis, the cut-off level of 24 pg/ml could predict the developmental delay with sensitivity and specificity of 96 and 92%, respectively.

**Conclusion:**

The IL-6 level and HIE grade are potential prognostic biomarkers for the determination of mortality and morbidity in asphyxiated neonates.

## Introduction

Despite advances in perinatal care, asphyxia is a major cause of mortality and permanent neurological as well as developmental complications in neonates (1). According to the World Health Organization (WHO), mild-to-severe asphyxia occurs in 3% (6.3 million cases) of newborns born in developing countries. It claims 840,000 lives and causes severe consequences in almost all survivors (2).

Perinatal asphyxia may result in multi-organ complications like respiratory distress syndrome, disseminated intravascular coagulation (DIC), subcutaneous fat necrosis, myocardial ischemia, adrenal hemorrhage, metabolic disorders, and acute tubular necrosis. Although most of the consequences are transient, asphyxia may cause hypoxic ischemic-encephalopathy (3). Its incidence is 1 to 4 in 1,000 live births (4) and may have consequences such as cerebral palsy, epilepsy, and learning disabilities (2, 5). Therefore, prediction of prognosis of asphyxia is a priority for both prevention and treatment of its complications (6).

Currently, the diagnosis of asphyxia is based on electronic fetal monitoring during labor, presence of meconium in the amniotic fluid, Apgar score, signs of HIE (7, 8), and multi-organ disorder within 72 hours after delivery. These clinical symptoms are supplemented with laboratory tests such as measuring fetal scalp blood pH, arterial blood gases testing, nucleated red blood cells, and biochemical markers like lactate, lactate dehydrogenase (LDH), creatine kinase (CK), neuron specific enolase, and several other proteins (9). The role of antioxidant-oxidant balance, heat shock protein, and interleukins (10) in prognosis of asphyxia is evaluated by several studies. 

Hence, determining the prognosis of asphyxia is considered a priority for the prevention and treatment of its complications (6). There is no correlation between current diagnostic criteria and patients’ prognosis, and in the absence of reliable markers (11), early diagnosis of HIE of asphyxia and its related brain damage is a challenging task in neonatal care (12). Recently, it has been reported that inflammatory cytokines contribute to the pathogenesis of ischemic brain injury (13). Increased level of IL-1 ß, IL-8, and IL-6 is reported in neonates who suffer from asphyxia (14). It is well-documented that IL-6 concentration in the cerebrospinal fluid (CSF) is associated with severe HIE, brain damage, and neurological outcomes (15). In addition, some studies reported increased levels of IL-6 and tumor necrosis factor-alpha (TNF-α) in the cerebrospinal fluid of infants with HIE (16). While both asphyxia and sepsis cause increased plasma level of IL-6 concentration, TNF-α level is more associated with sepsis (16). 

Serum IL-6 level has been reported to increase within the first 24 hours after hypoxic ischemia (16). Therefore, measuring the IL-6 level in the umbilical cord blood and peripheral blood serum has been reported as a relatively simple method to predict brain damage and other adverse consequences caused by asphyxia (3, 17). In the present study, serum level of IL-6 and other clinical and laboratory results were evaluated as biomarkers for prognosis of perinatal asphyxia, which is a less invasive approach. Hence, the current study aimed to evaluate the prognostic value of interleukin-6 (IL-6) and hypoxic-ischemic encephalopathy (3) grade in the prediction of mortality and developmental status of neonates affected by prenatal asphyxia**.**

## Materials & methods

**Selection of participants**

The study protocol of this observational (prospective cohort) research is approved by the Ethics Committee of the Mashhad University of Medical Sciences (IR.MUMS.MEDICAL.REC.1397.631), Mashhad, Iran. The study was conducted from Jan. 2013 to Oct. 2017 on eligible patients admitted to the Ghaem Hospital, Mashhad, Iran. We also considered a nested case-control design that intended to evaluate asphyxiation in infants. In addition, the current study intended to evaluate the developmental delay (DD) in a two-year follow-up period. Finally, we analyzed the IL-6 levels as a predictor of developmental delay.

Neonates who had at least two of the following criteria were considered eligible:

1. Fetal distress (late deceleration, lack of heart rate variability, and FHR< 100); 

2.Thick meconium-stained amniotic fluid and hypotonia or bradycardia or respiratory distress immediately after birth; 

3. Apgar score below 4 within the first minute or an Apgar score below 7 within the first 5 minutes. 

4. Those who required cardiopulmonary resuscitation for more than 1 minute using oxygen and intermittent positive pressure ventilation (IPPV); 

5. Blood pH < 7.2 and base deficit (18) < 12. 

Initially, the objectives of the study were explained to all potential participants and, then, if agreeing, written informed consent was obtained from their parents or guardians. If there were evidence of fetal distress or meconium release, parents were informed antenatally. Otherwise, parents were approached while infants were treating for asphyxia. It worth noting that we performed no extra intervention. Therefore, the parents were only asked to allow the research team to collect the necessary data from their infants and also to cooperate during the follow-up period. For those cases that the consent was obtained within the first hour after the birth, the IL-6 was measured in the serum sample collected for routine chemistry analyses. Exclusion criteria were congenital malformations, metabolic disturbances, sepsis, congenital or perinatal infections, and maternal chorioamnionitis. 

**Clinical Examination**

A comprehensive clinical examination was performed on all subjects at birth as well as 3 and 7 days after the birth. It is worth noting that all examinations were performed by a neonatologist. According to the Sarnat staging scale, hypoxic-ischemic encephalopathy (3) was classified as mild (Grade 1), moderate (Grade 2), or severe (Grade 3) (19). Mild HIE (Grade 1) was defined as being hyperalert, irritable, and hyperreflective, but no seizure in the first 24 h after the birth. While lethargy, hypotonia, hyporeflexia, myosis, and seizure were considered as symptoms of moderate (Grade 2) HIE. Eventually, severe (Grade 3) HIE was defined as apnea, flaccid, frequent seizure, and coma.

All eligible infants were followed up for two years and their development status was evaluated four times with 6-month intervals using the Denver Developmental Screening Test II (DDST-II). This test is designed to evaluate functions related to personal and social, fine motor and adaptive, language, as well as gross motor skills (20). Abnormal development was defined as mild, moderate, or severe if the child had a delay in one, two, three, or more domains in the DDST-II test, respectively. Eventually, asphyxiated children were divided into two groups of normal and abnormal development, based on the DDST-II screening. 

**Laboratory tests**

Blood samples (1-2 ml) were collected from the asphyxiated infant using umbilical blood sampling immediately after the birth. The serum was separated by centrifugation and stored at -70°C before analysis. Serum IL-6 concentration, pH, BE, pCO2, pO2, and O2 saturation were measured in all Samples. A Sysmex KX-21 Automated Hematology Analyzer (Sysmex, Kobe, Japan) was used for cell counting. Arterial blood gas (ABG) analysis was performed using a GEM Premier 3000 (Instrumentation Laboratory, Bedford, MA). Serum urea and creatinine were estimated using a BT 3000 chemistry analyzer (Biotechnica, Rome, Italy). Serum IL-6 levels were measured in duplicate using an enzyme-linked immunosorbent assay (ELISA) kit (Bender Med Systems, GmbH, Vienna, Austria). 

***Statistical analysis***

Data were analyzed using SPSS version 19 (Apache Software Foundation, and Chicago, IL, USA) and GraphPad Prism (GraphPad Software, San Diego, CA). The findings are described using mean ± standard deviation (SD). In order to evaluate the association between diagnostic markers (i.e. pH, the severity of asphyxia, and IL-6), regression models were developed. The sensitivity and specificity of the IL-6 testing concerning the determination of prognosis of asphyxia were evaluated. Statistical significance was considered when p-value<0.005. 

## Results

**Clinical and Laboratory Findings**

Initially, 47 asphyxiated neonates were recruited, but 16 were removed, 9 due to diagnosis of congenital infections, 2 because of inappropriate sample collection, and 5 (two with grade 2 and three with grade 1 HIE) discontinued during the follow-up period. In the present study, the loss to follow-up rate was less than 20%. This means that the recommended follow-up rate of 60-80% was met. Hence, it can be argued that the probability of bias has declined significantly. Finally, 38 children completed this study, in which 13 (34%) had normal and 25 (66%) abnormal development.

For those in the normal and abnormal groups, the mother age was 25.30±4.90 and 21.50±9.18 years, respectively, which were not significantly different (*P* > 0.2). As presented in Table 1, there was no significant difference between these two groups concerning the frequency of pregnancy complications (i.e. diabetes, preeclampsia, eclampsia, hypertension) and the type of delivery. Of 13 children with abnormal development, 6 (46%) did not have a history of HIE, 6 (46%) had HIE grade 1, and one infant (8%) had grade 2 HIE. However, all infants in the abnormal development group had a history of HIE; 3 infants (12%) grade 1, 10 (40%) grade 2, and 12 (48%) grade 3. Nearly 75% of those in this group had a history of the need for mechanical ventilation. Finally, 5 cases (20%) suffered from mild, three (12%) moderate, and three (12%) severe developmental delay, and 14 children (56%) died during the study period (Table 1).

**Table 1:** Comparing the maternal and neonatal clinical variables between the study groups

**Groups**	Normal Development 13, (34%)	Abnormal Development 25, (66%)	*p*-Value
**Variables**			
**History of pregnancy complications** ^*^			0.321
Yes No	6 (67) 3 (33)	9 (45) 11 (55)	
**Type of delivery**			0.923
Normal vaginal delivery Cesarean section	5 (41) 7 (59)	11 (44) 14 (56)	
**History of mechanical ventilation after birth**			0.001
No Yes	11 (100) 0 (0)	6 (25) 18 (75)	
**HIE degrees**			0.001
Without HIE HIE grade 1 HIE grade 2 HIE grade 3	6 (46) 6 (46) 1 (8) 0 (0)	0 (0) 3 (12) 10 (40) 12 (48)	
**Severity of developmental delay**			0.001
Normal developmental Mild delay Moderate delay Severe delay Death	13 (100) 0 (0) 0 (0) 0 (0) 0 (0)	0 (0) 5 (20) 3 (12) 3 (12) 14 (56)	

* Pregnancy complications (i.e. diabetes mellitus, pre-eclampsia, eclampsia, and hypertension)

There was no significant difference concerning the neonatal weight, fifth minute Apgar score, duration of hospital stay, white blood cell and platelet counts (WBC and PLT), sodium, potassium, Base Excess, HCO_3_^-^, and *p*CO_2 _between the study groups (*p*>0.05, Table 2). However, these two groups were different concerning the severity of HIE (*p*<0.001), serum urea ((*p*<0.001), and creatinine (*p*<0.01) as well as arterial pH (*p* <0.005), all measurements were performed one hour after the birth. ​​

**Table 2:** Comparing clinical and laboratory parameters between the study groups

	**Normal Development (n=13, 34%)**	**Abnormal Development (n=25, 66%)**	***p-value***
**Weight (g) **	3432± 1156	3362±1007	0.872
**One-minute Apgar **	5.1± 2.1	4.1±1.6	0.136
**Five-minute Apgar **	6.8 ± 2.8	5.6 ± 1.7	0.116
**Length of stay (day) **	11.00 ± 2.4	13.3 ± 3.1	0.642
**IPPV (h) **	54.8 ± 15.1	85.6 ± 21.8	0.003
**WBC (x1000)**	15.3 ± 5.8	23.1 ± 10	0.065
**Plt (x1000)**	218±36	164± 75	0.362
**Urea (mg/dl)**	18±8	43±25	0.001
**Creatinine (mg/dl)**	0.5 ± 0.2	1 ± 0.5	0.007
**pH **	7.25 ± 0.1	7.00± 0.2	0.001

The hematological and chemistry analyses were performed within the first hour after the birth. The values are presented as Mean±SD. IPPV: Intermittent Positive Pressure Ventilation, WBC: White Blood Cells, Plt: Platelet, BE: Base Excess.

**Figure 1:**
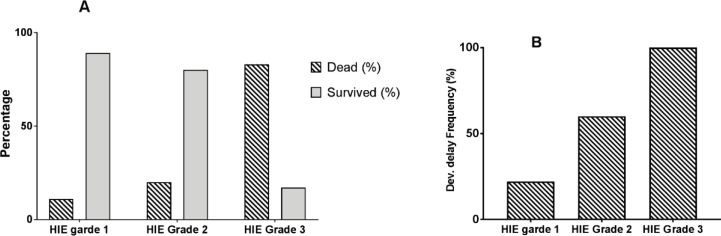
Effects of HIE grade variation on survival rate (panel A) and prevalence of developmental delay in the survivors (panel B), HIE: hypoxic-ischemic encephalopathy; Dev Delay: percentage of survivors with developmental delay

The effects of HIE grade variation on survival (panel A) and prevalence of developmental delay (panel B) are shown in Figure 1. While only 11% of neonates with HIE grade 1 died and 22% of the survivors had mild developmental delay, about 27% of the infants with grade 2 eventually died and 60% of the survivors had developmental delay. Only 17% of those who were suffering from HIE grade 3 survived, in which all had developmental delay. There was a direct correlation between the severity of HIE and the prevalence of both developmental delay (*p*<0.001) and mortality rate. The correlation rates between HIE grade with mortality rate and developmental delay were 84 and 99%, respectively. 

**Correlation between IL-6 Concentration and HIE grade**

**Figure 2:**
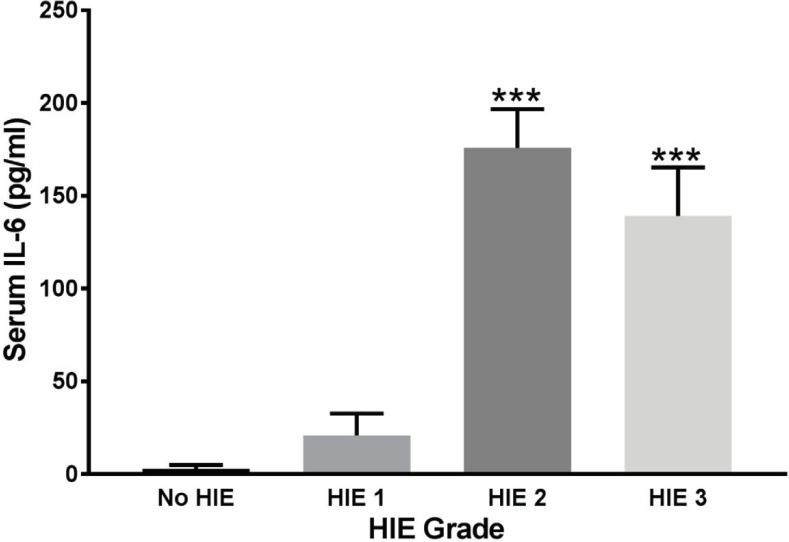
Interleukin-6 concentration in the asphyxiated neonates with different grades of hypoxic-ischemic encephalopathy (3). Data are presented using the mean values of the IL-6 concentration ± SEM. *** Indicates a statistically significant difference (P<0.001) in IL-6 concentration in NO HIE group and those with HIE grades of 2 and 3

IL-6 concentration in the asphyxiated neonates with different grades of HIE is shown in Figure 2. According to the findings, the mean level of IL-6 was increased from 2.7 in the control group (not affected HIE) to 29, 175, and 136 ng/ml in those with HIE grades of 1, 2, and 3, respectively. Hence, HIE grade 1 did not significantly change the IL-6 level ((*p>* 0.1). It also suggests that IL-6 concentration may reach its maximum in neonates with HIE grade 2. Therefore, there was no significant difference concerning the IL-6 concentration between those with HIE grades of 2 and 3 (*p>* 0.2). 

**Correlation between IL-6 Concentration and Developmental Delay**

**Figure 3:**
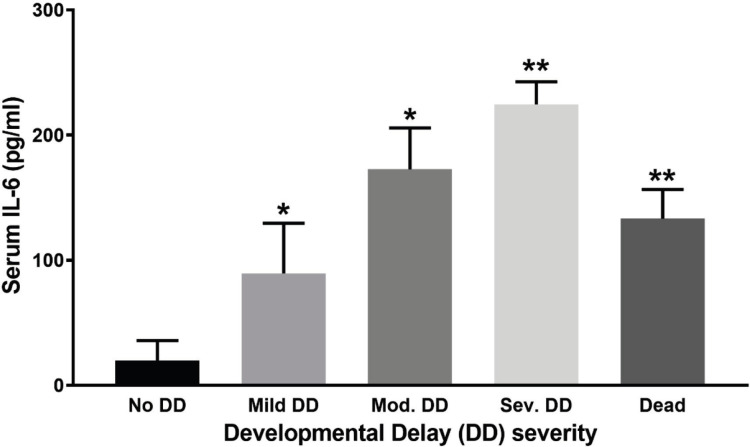
Comparing the mean IL-6 concentrations among participants with different outcomes, The study groups included an observational category with no development delay (No DD); Mild DD: Mild developmental delay; and Mod. DD: moderate developmental delay; Sev. DD: Sever developmental delay; Dead: died during the two-year follow-up period. Data presented as the Mean values of the IL-6 concentration ± SEM. * and ** indicate a statistically significant difference in IL-6 concentration with p-values less than 0.05 and 0.01, respectively

As shown in Figure 3, there was a statistically significant difference concerning the mean IL-6 concentrations between the control group with the normal outcome (no developmental delay) and those with different levels of developmental delay. While, initially, the IL-6 level was 4 (No developmental delay), it increased up to 50, 173, and 225 ng/ml in those with mild, moderate, and severe developmental delay, respectively. There was a direct correlation (96%) between IL-6 level and the severity of the developmental delay. Further analysis showed that the cut-off levels of 25, 62, and 101 pg/ml for predicting mild, moderate, and severe developmental delay. Serum IL-6 level had a median of 112 and 42 among deceased participants who had HIE grades of 2 or 3. 

**Figure 4:**
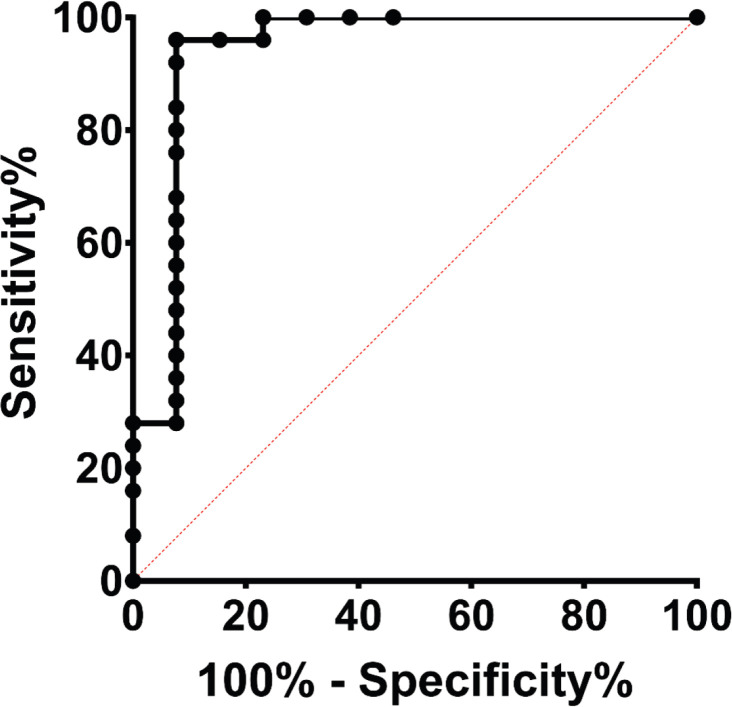
ROC curve analysis of the IL-6 levels for prediction of developmental delay. The level of IL-6 was measured in the asphyxiated infant using umbilical blood sampling immediately after delivery. Developmental delay was evaluated using the Denver Developmental Screening Test II (DDST-II) after two years of follow-up

## Discussion

To improve the management procedures, many attempts have been made to accurately predict the prognosis of asphyxiated neonates by combining the results of clinical examinations with laboratory tests. Similar studies mentioned HIE grade, 5-minutes Apgar score, need for ventilation, initial arterial pH, and the severity and duration of antepartum metabolic acidosis as important factors in the determination of prognosis of asphyxiated infants (21). 

According to the findings, there was no significant difference between the asphyxiated newborn with normal and delayed development concerning the number of leukocytes and platelets, which is consistent with a similar study (22). However, the results showed that 2 years old children with developmental delay had higher neonatal serum urea and creatinine level as well as lower initial arterial pH, which is consistent with other studies (23). According to the previous studies, the fifth minute Apgar score and pH were lower in the asphyxiated neonates compared to the healthy newborns (24). The severity of the HIE was an important factor in the prediction of mortality rate and developmental delay in the survivors. The mortality rate was 11, 27 and 83% among infants with HIE grades of 1, 2, and 3, respectively. The overall rate of asphyxia-associated death has been reported to be about 10-30%, and the prevalence of neurological complications among survivors ranged from 15 to 45% (21).

In the same vein, another study reported that the APGAR scores were mild to normal in 68%, moderate in 22%, severe in 10% of asphyxiated neonates. Additionally, the SARNAT stages were I in 25%, II in 52%, and III in 24% of asphyxiated cases. Mortality rates were 66.7% in SARNAT stage III, 22.2% in SARNAT stage II, and 11.1% in SARNAT stage I (25). However, 50-89% of those with grade 3 eventually would die, along with severe consequences for the development of survivors. While only 9.8% of children with an HIE grade of 1 had mental retardation; all neonates who suffered from grades of 2 and 3 HIE ended up with mental retardation, borderline IQ, and average IQ (26). Furthermore, it has been reported that 25-30% of infants with moderate HIE are predisposed to developmental abnormality or even death (27). 

In earlier studies, the combination of serum levels of IL-6 (>41 pg/ml) along with moderate to severe HIE has been suggested as a prognostic biomarker for diagnosis of developmental delay and even early death (27). The current study provided more details about the merits of combining IL-6 concentration and HIE grade in the determination of asphyxia-related mortality and morbidity rates. Regardless of the first hour IL-6 concentration, 83% of the neonates with HIE grade of 3 would die within two years and all survivors will probably develop severe developmental delay. IL-6 is highly valuable in the determination of the prognosis of survivors. For neonates with an IL-6 level of >25, 62, and 101pg/ml, the child has mild, moderate, and severe developmental delays at the age of 2 years, respectively.

There is a controversy regarding the role of IL-6 in the pathogenesis of brain damages caused by asphyxia. The rise in IL-6 level may support the possible role of this cytokine in the pathogenesis of hypoxic-ischemic brain injury (16). However, it has been suggested that IL-6 might be released as a protective response after hypoxic-ischemic brain injury and is involved in the repair process in the sub-acute stage of HIE. On one hand, the findings of the present study revealed a direct correlation between IL-6 concentration and the severity of developmental delay, which supports its destructive role. On the other side, those who deceased had a lower level of IL-6, mainly as a consequence of HIE, which suggests its possible protective effects. This study demonstrated hypoxia stimulates IL-6 production via activation of MAP kinase (Mitogen-activated protein kinase), HIF-1 (hypoxia-inducible factor-1), and NF-kappa B (nuclear Factor-kappa B) (28, 29). However, cancer-related studies provided evidence that hypoxia may suppress the immune system (30). Our results showed a decrease in the IL-6 level by increasing HIE grade from 2 to 3, which suggests that while mild to moderate hypoxia can induce IL-6 expression. In addition, there was a negative association between immunosuppressive effects of severe hypoxia and IL-6 expression. 

Due to practical difficulties in the recruitment and follow-up stages, a limited number of patients were included in some groups, which is a common problem in such clinical studies. However, it seems that the strong correlation between the variables, resulted from the prognostic power of these biomarkers, could compensate for the adverse effects of that limitation. More comprehensive studies may result in establishing a combination of HIE grade and IL-6 as prognostic biomarkers for neonatal asphyxia. 

## Conclusion

This study revealed more details and suggested cut-offs for HIE grades and IL-6 levels, as potential prognostic biomarkers for mortality and morbidity caused by neonatal asphyxia. When followed for two years, neonatal IL-6 concentrations above 24 nl/L combined with HIE grades of 2 and 3 and acidosis indicated a high mortality rate and developmental delay among survivors. Therefore, these three parameters may have a crucial role in the identification of those asphyxiated neonates who need early intervention to alleviate asphyxia-related developmental delay.

## Author’s Contribution

Gholamali Maamouri, Maryam Zakerihamidi conceptualized and designed the study, drafted the initial manuscript, and approved the final manuscript as submitted.

Baratali Mashkani, Abbas Boskabadi, Shahin Mafinejad, and Rahelah Faramarzi designed the study and carried out the initial analyses, reviewed and revised the manuscript, and approved the final manuscript as submitted.

Hassan Boskabadi, Ezzat Khodashenas, Fatemeh Bagheri, Elaheh Heidari, and Forough Rakhshanizadeh carried out the initial analyses, reviewed and revised the manuscript, and approved the final manuscript as submitted.

Maryam Zakerihamidi designed the data collection instruments, and coordinated and supervised data collection at two of the four sites, critically reviewed the manuscript, and approved the final manuscript as submitted.

All authors approved the final manuscript as submitted and agree to be accountable for all aspects of the work.
